# Aromatase Inhibition Ameliorates Decreased LH Output Found in Obese Women

**DOI:** 10.1007/s43032-019-00105-5

**Published:** 2020-01-06

**Authors:** Kelsey Jones, Sarah Ryan, Nichole E Carlson, Justin Chosich, Andrew P. Bradford, Nanette Santoro, Alex J Polotsky

**Affiliations:** 1 Department of Obstetrics and Gynecology, University of Colorado School of Medicine, 12700 E. 19th Ave, P-15-3450A, Aurora, CO 80045 USA; 2 Department of Biostatistics and Informatics, University of Colorado School of Public Health, Aurora, CO 80045 USA

**Keywords:** Obesity, Letrozole, Aromatase inhibitor, FSH, LH

## Abstract

In obese ovulatory women, serum luteinizing Hormone (LH) and follicle stimulating hormone (FSH) are lowered compared with normal weight women. This relative hypogonadotropic hypogonadism represents a potential etiology for overall decreased fertility in obesity. The objective was to determine if administration of an aromatase inhibitor (AI) to ovulating obese women would normalize LH and FSH by interrupting estradiol negative feedback. Letrozole (2.5–5 mg) was given daily to 22 women, 12 obese and 10 normal weight, for 7 days. On the last day of administration, 8 h of blood sampling was done every 10 min before and after a bolus of GnRH at 4 h. We obtained data from 21 ovulatory women (10 normal weight and 11 obese) who had undergone a similar protocol of frequent blood sampling but no aromatase inhibitors (AI) treatment. Serum LH and FSH levels and pulse characteristics were measured. Treatment with AI only significantly affected obese women. Further, in women with obesity, LH secretion, prior to the GnRH bolus, was significantly higher in AI treated compared with non-treated (*p* = 0.011). AI treatment doubled LH pulse amplitude in obese women (*p* = 0.004). In response to aromatase inhibition, LH secretion in ovulatory women with obesity is increased and similar to levels found in untreated normal weight women. The increase in LH pulse amplitude indicates that the AI effect is mediated at the level of the pituitary. Our results suggest that the hypogonadotropic phenotype of simple obesity is subject to modulation by interruption of estradiol negative feedback.

## Introduction

Obesity makes it more difficult for women to conceive, whether through natural or assisted methods. Obesity is known to affect the hypothalamic-pituitary-ovarian axis, oocyte quality, and overall fertility [1–3]. With the prevalence of obesity estimated at over 50% in developed countries, it is important to understand pathophysiology and possible avenues for intervention [3].

Luteinizing hormone (LH) is produced by the pituitary gonadotropes and is essential for ovulation. Cellular mechanisms involve the theca cells in the ovary, whereby products of these cells permit the granulosa cells to produce estrogens. Estrogens provide negative feedback on pituitary LH secretion through the mid-follicular phase. Eventually, the buildup of estrogen is so great that an LH surge follows and induces ovulation [4]. In an ovulatory obese woman, both LH and FSH are consistently demonstrated to be reduced with a corresponding reduction in estrogen and progesterone production by the ovary after ovulation. This relative hypogonadotropic hypogonadism represents a potential etiology for overall decreased fertility in ovulatory obese women [5].

Aromatase inhibitors (AI) have been used to resolve hormone imbalances in women without PCOS. Aromatase is a microsomal cytochrome P450 hemoprotein known to carry out the key steps of converting androstenedione to estrone and testosterone to estradiol. [6, 7] It is found in many different types of tissues including brain, breast, placental, ovarian, testicular, endometrial, skin, bone, and fat tissues [7]. Inhibitors of the aromatase enzyme have been shown to aid in regulation of LH levels and pulse amplitude. [6, 8, 9] A study by Bayar et al. showed that AI administration provided an 81% ovulation rate in anovulatory participants and an overall 9% pregnancy rate. [10] We have previously shown that by interrupting the initial negative feedback of estrogen on LH in the early follicular phase, LH levels are increased during the menstrual cycle [9]. By increasing LH during this stage, one increases the chance of having normal levels of downstream hormones. It is possible that since FSH follows the same secretion pattern as LH and is also secreted from the pituitary, these levels could rise as well [7].

The objective of the current report is to look at the ability of letrozole, an aromatase inhibitor, to normalize the differing hormone profiles of obese and normal weight (NW) women. Letrozole has a relatively short half-life, is a reversible inhibitor, and can be taken in pill form [7, 8]. In a previous pilot report, we administered letrozole, with 4 h of frequent blood sampling to five normal weight women. GnRH stimulation, as part of the protocol, was not previously reported [9]. We hypothesize that giving letrozole to ovulating obese women will shift their hypogonadotropic phenotype to favor increased levels of LH and FSH by way of interrupting estrogen negative feedback.

## Materials and Methods

For this study, women ages 18–40 with a normal BMI of 18–25 kg/m^2^ (NW; *N* = 10) and women with a BMI above 30 kg/m^2^ (obese; *N* = 12) were recruited and treated with letrozole in the early follicular phase. All were screened for PCOS, determined by the presence of oligomenorrhea, as outlined by the NIH. All of the participants had regular menstrual cycles within the 25–35 day range. Other inclusion criteria included age 18–40 at enrollment, regular menstrual cycles of 25–35 days, no presence of chronic disease interfering with reproductive hormones, normal TSH and prolactin, and no use of medications that would interact or disrupt reproductive hormones. Women who participated in excessive exercise (more than 4 h/week) were excluded.

### The Protocol

Starting on cycle days 2–5, letrozole was given for a total of 7 days, based on body surface area. Therefore, six of the obese women received 5 mg while the rest received 2.5 mg. After 7 days of treatment, participants underwent frequent blood sampling every 10 min for 6 h with a bolus of GnRH (75 ng/kg) given at 4 h prior to the end of the study visit. All but one participant from this study collected daily urine samples over the course of this menstrual cycle, which were measured for levels of FSH, LH, PDG, and E1c [6].

### Historical Controls

Untreated normal weight and obese controls were obtained from another study conducted by our group [ 11]. There were 21 participants (*N* = 10 NW, *N* = 11 obese) who had frequent blood sampling every 10 min for 8 h with a bolus of GnRH (85 ng/kg) given 2 h prior to the end of each study visit in the early follicular phase. This protocol was done both prior to and after transdermal estradiol priming. In this paper, we are only using the untreated baseline data as a comparison group. Participation criteria for this group was the same as above except for the following: age range was 18–42 years; regular menses was defined as every 25–40 days; in addition to normal TSH and prolactin, blood counts had to be normal; PCOS was ruled out in the same manner as our study; participation was prohibited if there was positive screen for protein C or other contraindication to exogenous estrogen; and smoking, hypertension, and use of medication affecting reproductive hormones, exogenous sex steroids, and attempting pregnancy were also means of exclusion. Participants were recruited from the University of Colorado Anschutz Medical campus as well as the Albert Einstein College of Medicine. All participants provided written informed consent. The Institutional Review Boards of Albert Einstein College of Medicine and the University of Colorado School of Medicine’s Combined IRB (COMIRB) approved the study.

In all studies, DELFA immunofluorometric assay (Perkin-Elmer) was used to measure LH and FSH. Inter-assay and intra-assay CVs: 4.8% and 5.4%, for LH and 6.3% and 4.2% for FSH. Daily urine samples were assayed for E1c and Pdg. Hormone concentrations were adjusted for glycerol and normalized to creatinine [12]. The E1c and Pdg levels were measured by ELISA and normalized to a 28-day cycle. The intra-assay and inter-assay CVs were 2.2% and 6.8%, respectively for E1c; and 2.3% and 4.5%, respectively for Pdg.

### Data Analysis

**Fig. 1:**
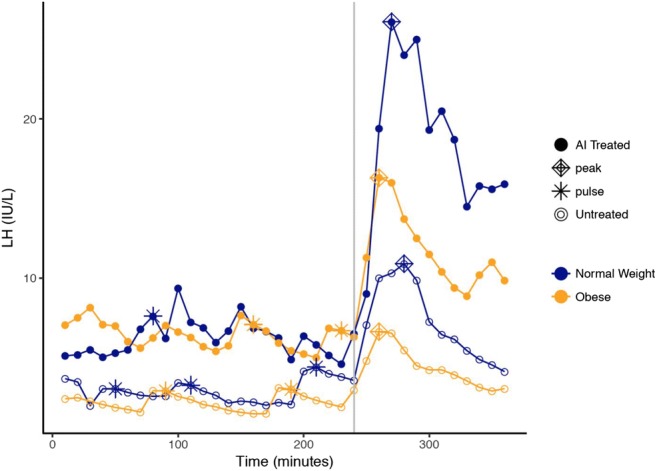
Hormone curves from subjects representative of AI treated (solid dots) vs. untreated (open dots), and normal weight (blue) vs. obese (orange). A bolus of GnRH was administered at 240 min, as indicated by the vertical gray line. Pulses are represented by asterisks; peaks are represented by diamonds

LH pulsatility was characterized using the first 4 h of data from each study period. LH pulse frequency and amplitude were computed for each individual using a modified Santen-Bardin pulse detection method that has been previously validated [13]. Post-GnRH administration, both LH and FSH mean serum level, peak level, area under the curve, and maximum response were calculated; representative LH hormone profiles illustrating these parameters, from normal weight and obese patients, in the control and AI-treated arms of the study are depicted in Fig. 1. These measures were computed using only the time of the bolus and 2 h afterwards to have equivalency across the two studies. The peak level was defined as the maximal hormone concentration after administration of the GnRH bolus, and the maximum response was defined as the arithmetic difference between the nadir prior to GnRH and the peak post GnRH, where the nadir is the average hormone level in the hour preceding GnRH administration. Outcomes were log (base-e) transformed to address potential skew.

Linear regressions assessed whether BMI influenced the effect of AI treatment on the outcomes. The linear contrasts were used to calculate pairwise differences between BMI and treatment groups. Outcomes that were log transformed were returned to their natural scale and presented as geometric means, with fold (or percent) change between groups. Results were considered significant at a significance level of 0.05.

## Results

AI-treated and untreated obese women had similar average age and BMI (*p* > 0.55; Table 1). AI-treated and untreated NW women also had similar average ages and BMI (*p* > 0.69; Table 1). Within treatment group, obese and NW women had similar average ages (*p* > 0.30; Table 1).

**Table 1:** Effect of treatment and obesity on demographics and hormone levels. Age, BMI, and pulse counts (indicated by *) are presented mean (95% CI) with group differences, as they were analyzed on their natural scale. All other variables were log-transformed prior to analysis, with results presented as geometric mean (95% CI) with fold changes between groups. Significant results are bolded

Variable	AI treated			Untreated			Overall *p* value of interaction	Fold change treated vs. untreated in NW group	Fold change treated vs. untreated in obese group
	Normal weight (*N* = 11)	Obese (*N* = 12)	Fold change	Normal weight (*N* = 10)	Obese (*N* = 12)	Fold change			
Age*	30.36 (27.06, 33.67)	30.50 (27.34, 33.66)	0.14 (*p* = 0.952)	29.40 (25.93, 32.87)	31.83 (28.67, 35.00)	2.43 (*p* = 0.301)	0.483	0.96 (*p* = 0.687)	− 1.33 (*p* = 0.551)
BMI*	21.32 (18.50, 24.15)	37.08 (34.38, 39.78)	15.76 (*p* < 0.001)	21.15 (18.19, 24.12)	37.64 (34.94, 40.35)	16.49 (*p* < 0.001)	0.793	0.17 (*p* = 0.934)	− 0.56 (*p* = 0.768)
Pre-GnRH									
LH pulse count*	2.36 (1.61, 3.12)	2.33 (1.61, 3.05)	− 0.03 (*p* = 0.954)	2.20 (1.41, 2.99)	2.00 (1.28, 2.72)	− 0.20 (*p* = 0.708)	0.820	0.16 (*p* = 0.764)	0.33 (*p* = 0.513)
LH mean amp. (IU/L)	2.42 (1.56, 3.75)	2.56 (1.66, 3.97)	1.06 (*p* = 0.854)	2.05 (1.30, 3.25)	1.01 (0.65, 1.56)	0.49 (*p* = 0.029)	0.088	1.18 (*p* = 0.599)	2.54 (*p* = 0.004)
LH mean level (IU/L)	6.94 (5.02, 9.60)	4.73 (3.47, 6.45)	0.68 (*p* = 0.091)	4.76 (3.39, 6.69)	2.65 (1.94, 3.61)	0.56 (*p* = 0.014)	0.527	1.46 (*p* = 0.112)	1.79 (*p* = 0.011)
FSH pulse count*	0.50 (− 0.13, 1.13)	0.45 (− 0.15, 1.05)	− 0.05 (*p* = 0.916)	1.10 (0.47, 1.73)	0.83 (0.26, 1.41)	− 0.27 (*p* = 0.531)	0.715	− 0.60 (*p* = 0.181)	− 0.38 (*p* = 0.362)
FSH mean amp. (IU/L)	1.40 (0.73, 2.69)	1.33 (0.76, 2.35)	0.95 (*p* = 0.909)	1.38 (0.90, 2.11)	1.12 (0.71, 1.78)	0.81 (*p* = 0.501)	0.759	1.02 (*p* = 0.967)	1.19 (*p* = 0.623)
FSH mean level (IU/L)	5.50 (4.24, 7.14)	5.12 (4.00, 6.57)	0.93 (*p* = 0.693)	4.65 (3.59, 6.04)	4.11 (3.24, 5.21)	0.88 (*p* = 0.477)	0.828	1.18 (*p* = 0.365)	1.25 (*p* = 0.200)
Post-GnRH									
LH mean level (IU/L)	14.11 (9.45, 21.08)	9.47 (6.45, 13.91)	0.67 (*p* = 0.155)	9.09 (5.97, 13.85)	4.87 (3.32, 7.16)	0.54 (*p* = 0.033)	0.572	1.55 (*p* = 0.134)	1.94 (*p* = 0.018)
LH peak level (IU/L)	20.50 (13.68, 30.74)	13.40 (9.09, 19.74)	0.65 (*p* = 0.133)	13.45 (8.80, 20.57)	7.11 (4.83, 10.48)	0.53 (*p* = 0.031)	0.597	1.52 (p = 0.155)	1.88 (*p* = 0.025)
LH time to peak (min)	277.2(265.4, 289.4)	269.7 (258.7, 281.1)	0.97 (*p* = 0.360)	269.7 (257.7, 282.2)	266.6 (255.8, 277.9)	0.99 (*p* = 0.708)	0.709	1.03 (*p* = 0.384)	1.01 (*p* = 0.697)
LH AUC	1650 (1104, 2465)	1146 (780, 1683)	0.69 (*p* = 0.192)	1070 (702., 1630)	585 (398., 859)	0.55 (*p* = 0.038)	0.548	1.54 (*p* = 0.140)	1.96 (*p* = 0.017)
LH max response (IU/L)	14.35 (8.84, 23.31)	8.56 (5.38, 13.63)	0.60 (*p* = 0.128)	9.73 (5.85, 16.19)	4.99 (3.14, 7.94)	0.51 (*p* = 0.057)	0.751	1.47 (*p* = 0.271)	1.72 (*p* = 0.104)
FSH mean level (IU/L)	7.13 (5.49, 9.25)	6.49 (5.06, 8.32)	0.91 (*p* = 0.602)	5.37 (4.13, 6.97)	4.59 (3.62, 5.83)	0.86 (*p* = 0.377)	0.805	1.33 (*p* = 0.129)	1.41 (*p* = 0.049)
FSH peak level (IU/L)	7.87 (6.08, 10.18)	7.39 (5.78, 9.45)	0.94 (*p* = 0.722)	6.83 (5.28, 8.83)	5.53 (4.37, 7.00)	0.81 (*p* = 0.229)	0.553	1.15 (*p* = 0.434)	1.34 (*p* = 0.093)
FSH time to peak (min)	312.9(295.7, 331.1)	313.5(297.1, 330.9)	1.00 (*p* = 0.959)	291.6 (275.6, 308.6)	296.6 (281.6, 312.3)	1.02 (*p* = 0.658)	0.784	1.07 (*p* = 0.082)	1.06 (*p* = 0.140)
FSH AUC	819(632, 1062)	746 (583, 955)	0.91 (p = 0.599)	640 (494, 829)	550(434, 697)	0.86 (*p* = 0.388)	0.817	1.28 (*p* = 0.181)	1.36 (*p* = 0.079)
FSH max response (IU/L)	2.47 (1.66, 3.67)	2.50 (1.68, 3.71)	1.01 (*p* = 0.971)	2.71 (1.83, 4.03)	2.15 (1.50, 3.08)	0.79 (*p* = 0.386)	0.530	0.91 (*p* = 0.738)	1.16 (*p* = 0.576)

### Differences in LH and FSH with AI Treatment in Obese

**Fig. 2:**
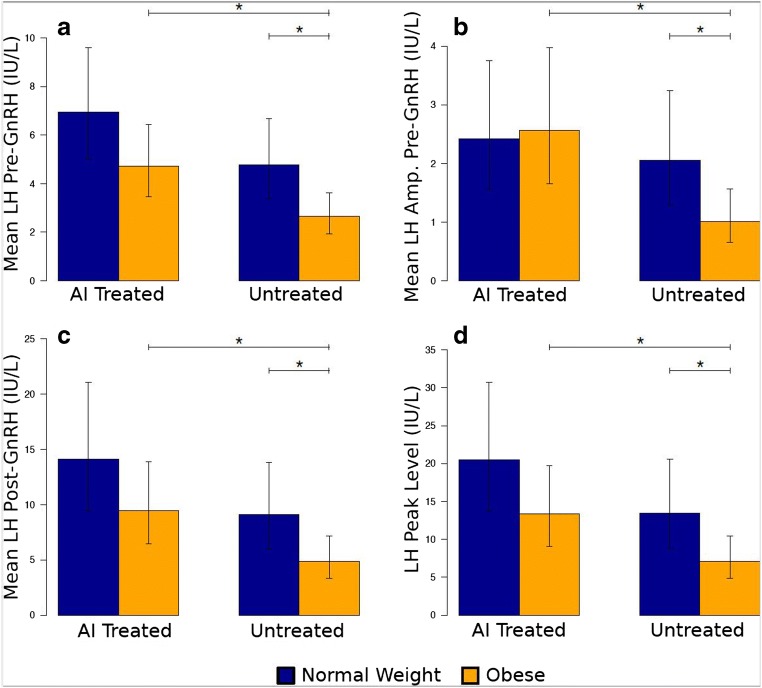
Differences in luteinizing hormone by AI treated vs. untreated, and normal weight vs. obese. Bar plots represent the geometric mean, with 95% confidence intervals (vertical lines); horizontal lines with an asterisk represent significant pairwise differences (*p* < 0.05). Amp, amplitude, calculated as described in materials and methods

Pre-GnRH stimulation, obese AI-treated women had higher mean levels of LH (4.73 IU/L, 95% CI 3.47, 6.45) compared with obese non-AI-treated women (2.65 IU/L, 95% CI 1.94, 3.61) (*p* = 0.011; Fig. 2; Table 1). These differences were maintained after GnRH stimulation (*p* = 0.018). The obese AI-treated and untreated women exhibited similar LH pulse frequencies (2.33 pulses/4 h, 95% CI 1.61, 3.05 vs. 2.00 pulses/4 h, 95% CI 1.28, 2.72, respectively; *p* = 0.51). However, the obese women treated with AI had, on average, larger pulses (2.56 IU/L, 95% CI 1.66, 3.97) compared with obese non-AI treated women (1.01 IU/L, 95% CI 0.65, 1.56).

Post-GnRH stimulation, AI-treated obese women had a higher mean peak LH of 13.40 IU/L (95% CI 9.09, 19.74) compared with 7.11 IU/L (95% CI 4.83, 10.48) in the non-AI-treated obese group (*p* = 0.025; Fig. 2). The LH AUC was also nearly double for the AI-treated obese women compared with the non-treated obese women (1146 IU/L, 95% CI 756, 1735 vs. 578 IU/L, 95% CI 382, 876, respectively, *p* = 0.024).

With the exception of average FSH after GnRH stimulation, FSH parameters did not differ in obese with AI treatment (*p* > 0.10). Average FSH levels after GnRH stimulation were higher in AI-treated obese compared with non-treated obese women (6.49 IU/L, 95% CI 5.06, 8.32 vs. 4.59 IU/L, 95% CI 3.62, 5.83, *p* = 0.049).

### Differences in LH and FSH with AI Treatment in Normal Weight

There were no statistical differences in LH parameters between NW AI-treated and non-AI treated women (*p* > 0.112) (Table 1; Fig. 2). Several interesting patterns were observed. AI-treated NW had higher average levels of LH (6.9 IU/L, 95% CI 4.0, 9.6) compared with normal weight non-AI-treated women (4.76 IU/L, 95% CI 3.39, 6.69), although statistical significance was not reached (*p* = 0.11). This pattern was consistent with post-GH stimulation (Table 1). With GnRH stimulation, average mean peak LH was 1.5 times higher in AI-treated NW women compared with non-treated NW women (20.50 IU/L, 95% CI 13.68, 30.74 vs. 13.45 IU/L, 95% CI 8.80, 20.57; *p* = 0.16). Pre-GnRH stimulation, the average LH pulse size also did not differ between AI-treated and non-treated groups in NW (2.42 IU/L, 95% CI 1.56, 3.75 vs. 2.05 IU/L, 95% CI 1.30, 3.25; *p* = 0.60).

There were no statistical differences in FSH parameters between the AI treated and untreated groups in women with normal weight (*p* > 0.082).

### Comparing Differences Between Groups

In the untreated groups, pre-GnRH, LH level and amplitude were significantly lower in obese women compared with NW (*p* = 0.014 and *p* = 0.029, respectively). Post-GnRH, LH mean, peak, and AUC were also significantly lower in obese women (*p* = 0.033, *p* = 0.031, *p* = 0.038, respectively) In contrast, there were no differences observed between normal weight and obese women in the AI-treated group (*p* > 0.091); this was true for both LH and FSH parameters (Table 1). However, obese women treated with AI had very similar LH parameters to untreated NW women, at baseline and post GnRH stimulation (Fig. 2; Table 1).

## Discussion

In this study, we examined LH and FSH in normal and obese women, with and without administration of the AI and letrozole and before and after GnRH stimulation. Mean LH is higher in both obese and normal weight women treated with letrozole, and all groups had an increase in mean LH after stimulation by GnRH. However, in NW women, the overall effects of AI were not statistically significant with respect to any LH or FSH parameters. In obese women, mean LH levels and pulse amplitude were significantly higher in response to AI treatment. Similarly, GnRH-stimulated LH and FSH levels were significantly greater in the AI treated group. Thus, obese women appear to selectively respond to AI treatment, with respect to gonadotropin levels.

FSH followed a similar pattern to LH (values were higher with administration of AI), but the percentage difference was smaller and not statistically significant. Although not statistically significant, it is possible that the changes in FSH could be clinically meaningful. Biologically, letrozole blocks estrogen biosynthesis, thereby reducing the negative feedback of estrogen on the hypothalamic/pituitary axis. Other work has shown that this allows for more secretion of FSH by the pituitary, which aids in follicular growth and development [14]. Thus, it remains possible that even small changes in FSH would increase fertility outcomes in obese women; however, a longitudinal study with pregnancy rates and outcomes would better answer this.

Obesity is known to alter gonadotropins [5], but we found that in response to letrozole, the baseline hormone profile of ovulating obese women and their response to GnRH are increased, restoring them to levels that are comparable to those observed in untreated NW women. Indeed, with GnRH stimulation, the mean LH value in AI-treated obese women exceeded the mean LH value of non-AI-treated normal weight women (Fig. 2; Table 1). Thus, AI treatment appears to rescue the hypogonadotropic phenotype of obesity.

Obesity can alter LH expression at the level of the pituitary, as demonstrated by Kucherov et al. [9] In our study we found a significant increase in LH pulse amplitude in the obese cohort after AI administration (Fig. 2; Table 1). Our results further support the pituitary as the site of action of aromatase inhibition, since the amplitude, rather than the frequency, increases after letrozole administration.

Strengths of this study include using the normal weight and obese control groups, tailoring the letrozole dose to body surface area, and looking directly at the pituitary to identify it as the site of LH control. Weaknesses include a relatively small sample size and observation period and the use of temporally separate control and intervention groups, precluding pairwise comparisons of individual participants. We acknowledge that potential effects on fertility are inferred, as the study did not examine pregnancy outcomes.

As the prevalence of obesity continues to grow in developed and developing countries, it will be important for physicians to educate their patients on how BMI affects their fertility and pregnancy outcomes. Modest weight reductions have been shown to increase ovulatory and reproductive success [15]. Future studies could focus on a design that incorporates administration of an AI, alongside monitored weight loss, to observe the effect of a combination of fertility therapies. This study shows the potential of letrozole to increase the chance of spontaneous pregnancy in obese women by normalizing their LH profiles.
